# Should small renal masses be managed expectantly?

**Published:** 2007

**Authors:** Manish Singla, Aneesh Srivastava

**Affiliations:** Department of Urology and Renal Transplantation, SGPGIMS, Lucknow, India E-mail: anees@sgpgi.ac.in

## SUMMARY

In this retrospective analysis the authors have studied the short-term outcomes of patients whose renal masses were managed expectantly (i.e., watchful waiting) to provide insight into the natural history of small renal tumors. This study also analyzed the effects of delayed intervention in patients who required intervention after a period of expectant management. A total of 43 patients with 46 renal masses underwent planned expectant management of enhancing solid or cystic (Bosniak IV) renal masses. A subset of these patients (13) who underwent eventual intervention was also examined. Outcomes with regard to age, gender, growth rate, subsequent interventions and clinical follow-up are reported. At a mean follow-up of 36 months 74% of patients had tumor growth with a mean (median) growth rate of 0.70 (0.35) cm per year. None of the patients had significant symptoms, disease progression or cancer-specific death. The 13 patients undergoing eventual intervention were younger than those who did not undergo intervention (56 vs. 72 years, respectively, *P* = 0.0006). Patients undergoing eventual intervention tended to have a higher tumor growth rate than those on continued observation (0.90 vs. 0.61 cm per year, respectively, *P* =0.1486). In patients undergoing eventual intervention 12 of 14 (87%) tumors were renal cell carcinoma. All were stage pT1 and 12 of 13 patients were alive after a mean follow-up of 41 months (one patient died of other causes 30 months after surgery). No patient had up-staging of disease secondary to delay.

## COMMENTS

In the present study small renal masses have been found to grow at an average rate of 0.70 and median of 0.35 cm per year, suggesting that smaller renal masses grow relatively slowly. The present study also attempts to identify factors that may be associated with more rapid growth rate. Interestingly, initial size and mode of presentation, symptomatic (28%) or asymptomatic did not correlate with growth rate in this population. Age was perhaps the strongest predictor of tumor growth, with younger patients (60 years old or younger) having more rapid growth rates.

There are several limitations to the present study. The follow-up period remains relatively short (approximately three years) which does not determine the long-term impact of a surveillance strategy. This is especially relevant for younger patients without significant co-morbidities. Accordingly, these results should not be taken as justification for observation of all renal tumors, especially in the younger, healthier patient.

This report represents a retrospective case series and not a prospective randomized surveillance trial. Thus, the findings are subject to potential selection biases that may affect the observed outcomes. For example, it is not surprising that those who underwent intervention had fewer co-morbidities and faster growth rates than those on continued observation, because such factors are likely to influence the decision to intervene. Similarly, it is conceivable that the co-morbid conditions (seen in 80% of patients in the surveillance group) could themselves potentially impact tumor biology and growth. Such questions remain unanswered in this and other retrospective reports.

Kassouf *et al.*[1] reported a growth rate of 0.49cm per year in their series of 24 patients. Chawla *et al.*[2] performed meta-analysis on natural history of renal masses and reported a mean growth rate of 0.28 cm yearly. This may be due in part to the presence of non-RCC pathologies and/or smaller tumor size in these studies. In another study of Gill *et al.*[3] 30% of the 100 tumors removed by laparoscopic partial nephrectomy were benign. Mean tumor size in this cohort was 2.8 cm. While it appears that the risk of malignancy is less in smaller lesions, the majority of these lesions are malignant with growth potential. 1.0% of patients in the meta-analysis population progressed to metastatic disease. No definitive protocol for radiographic follow-up of renal lesions exists since it is not the gold standard of therapy. With regard to the interval of imaging the authors believe that it is prudent to have more frequent follow-up during the first 24 months.

Watchful waiting for renal masses is an appropriate option for select patients, especially those with competing co-morbidities. Delayed intervention does not appear to adversely impact pathological outcomes. Younger patients may be more susceptible to rapid tumor growth and should be treated aggressively.
